# Predicting mental health of prisoners by artificial neural network

**DOI:** 10.37796/2211-8039.1031

**Published:** 2021-03-01

**Authors:** Elahe Allahyari, Mozhgan Moshtagh

**Affiliations:** 1 Department of Epidemiology and Biostatistics, School of Health, Social Determinants of Health Research Center, Birjand University of Medical Sciences, Birjand, Iran; 2 Social Determinants of Health Research Center, Faculty of Health, Birjand University of Medical Sciences, Birjand, Iran

**Keywords:** general health of prisoners, socioeconomic capital, age, life expectancy, hope of acceptance, prisoners, artificial neural network

## Abstract

**Introduction:**

Maintaining and improving prisoners’ health and their rehabilitation can be effective steps towards eliminating health inequalities and approaching the UN’s Sustainable Development Goals. Accordingly, identifying protective factors and health barriers of this vulnerable group and changing the prison into an environment that can deliver health interventions tailored to the needs of inmates can provide the basis for attaining justice in health.

**Purpose:**

This study builds on an artificial neural network model to determine the effect of demographic, psychological, criminological, and physical activity factors on prisoners’ general health.

**Methods:**

The study detected the patterns between variables using a neural network with nine inputs and one output. To determine the neural network with the minimum sum of squared errors, we evaluated the performance of all neural networks using varying algorithms and numbers of neurons in the hidden layer. For this purpose, the analysis of the data of 149 prisoners aged between 16 and 61 years was performed using SPSS-22 software.

**Results:**

The optimal neural network model was useful in predicting prisoners’ general health. In this model, the variables of occupation, life expectancy, age, and hope of acceptance were identified as the first most significant factors with 19.25, 17.45, 15.98, and 15.16 percentages, respectively, whereas the cause of incarceration, education level, type of drug misuse, and physical activity were the second most important factors with 8.82, 8.38, 7.91, and 7.04 percentages, respectively.

**Conclusion:**

Experiencing psychosocial pressures related to incarceration is more severe for the marginalized and disadvantaged individuals, persons in very young or old age ranges, and those with no history of incarceration, which can increase the likelihood of impaired health for these inmates. Consideration of the prisoners’ needs in proportion to their characteristics and provision of emotional and spiritual support of the inmates, especially in the early stages of incarceration, can help shape an effective adjustment process and select appropriate and efficient strategies.

## 1. Introduction

Eliminating health inequalities is one of the United Nations’ Sustainable Development goals. Alongside this, it is highly important to maintain and to promote the health and rehabilitation of inmates while incarcerated [1]. According to United Nations estimates, the number of people imprisoned worldwide is more than 10 million or, otherwise, 144 per 100,000 [1]. This vulnerable population does not enjoy a good level of health because of their exposure to psychosocial stress at different stages of life or due to their individualistic weaknesses and restrictions [2]. Most prisoners come from poor socioeconomic classes with insufficient material resources (money and healthy food) and health (care and medication) before they are imprisoned [1, 2]. On the other hand, difficult living conditions in prison such as limited physical space and over crowdedness, inadequate light and ventilation, poor sleep, exposure to violence, high prevalence of infectious and uncommunicable diseases among inmates, and separation from family and friends can pave the grounds for the incidence or exacerbation of various physical and mental diseases or substance and alcohol abuse in this group [1, 3–8].

Concerns about shame, stigma, and social pressures associated with the history of incarceration and the negative consequences on family, future occupation, housing, and family responsibilities are secondary stressors that prisoners experience in addition to the primary stresses experienced before and during incarceration [9–12]. Enduring these psychological pressures can lead to a deteriorated physical and mental health of prisoners and even their families [11, 13, 14]. Depression, antisocial personality disorder, substance abuse, self-harm, and suicide are more common among inmates worldwide than in the general population [1, 3, 15]. Prisoners also suffer from non-communicable and chronic stress-related illnesses that can result in many negative consequences and incur substantial economic and health costs for the families of prisoners and society in large [16]. Having a positive attitude or optimism toward the future, as well as perceived social support or a sense of acceptance and support from others can play a beneficial role in improving life quality and promoting physical and mental health. They can help one maintain his/her sense of well-being and physical and mental health by adapting to problems and stress [17–19].

It is important to examine the general health of prisoners to prevent these problems and alleviate their consequences. Improved health status and rehabilitation of prisoners is beneficial from both individual and social dimensions. It can reduce the direct consequences of the disease and affect the quality of life of the individual him/herself, prison staff, prisoners’ families, and the society after the prisoner is released. Hence, the prevention, diagnosis, and treatment of physical diseases and mental disorders can serve to achieve these goals. The research site is Qaen Prison, located in eastern Iran, near the Afghan border. The total area of the prison is 2,420 square meters, consisting of four sections, of which three are allocated to men: the general crime section (630 square meters, 94 prisoners), open or workers section (590 square meters, 160 prisoners), and drugs offenses section (850 square meters, 119 people). Prisoners are monitored, screened, and receive health packages for 3 to 7 days at the beginning of the entry. During their stay in prison, inmates can receive free medication and medical care (one general practitioner, one psychiatrist, one psychologist, two social workers, and three nurses).

### 1.1. Purpose

In this line, an assessment of prisoners’ health status can provide a conscious awareness of health needs and health-related inequalities between different groups. Such awareness can facilitate the approach to justice in health and may be conducive to identifying contributing factors in prisoners’ health and changing the prison into an environment that can deliver health interventions tailored to the needs of more vulnerable groups [1, 20, 21]. Therefore, it is necessary, first, to determine the extent to which different factors affect prisoners’ general health so that by alleviating these barriers, a step can be taken to promote prisoners’ general health and achieve the goals of justice in health. However, since complicated patterns govern the relationships between variables in human studies, it is important to use appropriate models. Artificial neural networks, inspired by biological neurons in the human brain, have wide applications in predicting, modeling, detecting, and classifying relational patterns between variables [22]. Compared to conventional models such as the response surface methodology (RSM), these models can identify patterns between variables with minimum sum of squared errors and high correlation coefficients and without the need for limiting assumptions such as normality in studies where relationships between variables are complicated [23–25]. The efficiency of ANN models has been proven to solve various problems in medicine, psychology, and even humanities [26, 27]. Therefore, in this study, we decided to use artificial neural network (ANN) modeling as one of the most frequently applied and powerful models in predicting and modeling general health of prisoners in order to determine the complex relationships between variables and the effect of demographic, psychological, criminological, and physical activity factors on prisoners’ general health.

## 2. Methods

The research site is Qaen Prison, located in eastern Iran, near the Afghan border. The total area of the prison is 2,420 square meters, consisting of four sections, of which three are allocated to men: the general crime section (630 square meters, 94 prisoners), open or workers section (590 square meters, 160 prisoners), and drugs offenses section (850 square meters, 119 people). Prisoners are monitored, screened, and receive health packages for 3 to 7 days at the beginning of the entry. During their stay in prison, inmates can receive free medication and medical care (one general practitioner, one psychiatrist, one psychologist, two social workers, and three nurses).

To perform this cross-sectional study, after the code of ethics (Ir.bums.REC.1398.343) was obtained, 149 imprisoned men lacking mental disorders, severe anxiety, and depression were selected by simple random sampling in 2019. At first, explanations about the research were offered to the subjects, and the participants signed informed consent forms. They were subsequently asked to complete the General Health Questionnaire (GHQ) and a preliminary checklist of information including age, education level, occupation, type of drug misuse, cause of incarceration, optimism or hope for the future, hope of acceptance, and physical activity.

### 2.1. General health questionnaire

First developed by Goldberg and Hiller, GHQ is one of the most well-known screening tools for mental disorders [28]. It is available in 12-, 28-, 30- and 60-item versions, although GHQ-28 is more frequently employed given its shortness and similar functionality to GHQ-60. Therefore, GHQ-28 was employed in this study. It includes four subscales, each containing 7 items. Items 1–7 relate to somatic symptoms and general health status; items 8–14 concern with anxiety/insomnia; items 15–21 are related to social dysfunction; and items 22–28 concern with depression. The tool requests people to rate their general health in the past month from 0 to 3 on a 4-point Likert scale. The questionnaire is standard and has been standardized in different societies and countries such as Iran. In the Persian version, the questionnaire was measured along with a parallel test Middlesex Hospital Questionnaire (MHQ), with a 0.55 correlation coefficient between the two tests [29]. The correlation coefficients between the subscales of this questionnaire and the total score ranged from 0.72 to 0.87, and the Cronbach’s alpha for all items was 0.90.

### 2.2. Data analysis

The neural network is a parallel processing procedure, formed by connecting simple computing units called neurons in the input, middle, and output layers. The input layer neurons are the independent variables, and the output layer neurons are the response variables. Therefore, the number of neurons in the middle layer, the type of connections between the neurons in this layer and other layers, and their binding functions are among the parameters that determine the structure of the neural network [30]. This study used a multilayer feed forward back-propagation neural network, which is a widely used and powerful neural network, for predicting and modeling prisoners’ general health. In the ANN model of this manuscript, life expectancy, acceptance expectancy, education, job, cause of incarceration, type of drug misuse, 30 minutes of physical activity per week, and age are the input variables score of GHQ questionnaire is the output variable. Information from all records were used in the training dataset with Adam optimizer and Initial Lambda, Initial Sigma, Interval Center, Interval Offset, and Maximum Training Epochs were 5e-7, 5e-5, 0, ±0.5, and automatically, respectively. For this purpose, 70% of the data were incorporated in the network training process and the remaining 30% for model evaluation.

The most commonly used functions, namely hyperbolic tangent or sigmoid, were employed to connect the input and middle layer neurons, and linear, hyperbolic tangent, or sigmoid functions were applied to connect the middle and output layer neurons [31]. Subsequently, appropriate functions were used to optimize the neural network by increasing the number of neurons in the middle layer. To avoid the effect of random allocation of weights and random correlations, each network was reiterated three times, and the average sum of squared errors was used as an appropriate index for model evaluation. A total of 105 network data were used in training the model, and the performance of the model was evaluated using the remaining 44 data. The performance of the optimal neural network model was presented in the form of graphs by comparing the results predicted by the model and the actual values. The graphs were drawn using standardized observes and expect values with the following formula:


(1) $$\text{y}=({\text{y}}_{\text{i}}-{\text{y}}_{\text{min}})/({\text{y}}_{\text{max}}-{\text{y}}_{\text{min}})$$

where *y* denotes the normalized value for *y_i_*, and *y_min_* and *y_max_* denote the minimum and maximum *y_i_* values, respectively. Finally, the significance level of the variables of age, education level (illiterate, primary/secondary school, high school and above), occupation (unemployed, worker, non-worker), life and acceptance expectancy, cause of incarceration (property, persons, and drugs), optimism and hope for the future (measured through the question “to what extent are you optimistic and hopeful about the future and the improvement of living conditions?”. The answers ranged by a four-point scale from very high to very low), hope of acceptance (measured using the question “to what extent do you hope to be accepted and supported by family, community, work environment, and friends?”. The answers ranged by a four-point scale from very high to very low), type of drug misuse (non-smoking, smoking, opioid), and 30 minutes of physical activity per week (Yes or No) were determined to predict the general health of prisoners. All analyses were performed using SPSS application tools (Statistical Product and Service Solutions) version 22 software.

## 3. Results

The study population consisted of 149 men aged 19 to 61 years with a mean age of 36.58 ± 8.73 years, most of whom (27.5%) had little life expectancy or no hope of acceptance (28.9%). Most of the subjects held a high school diploma (49%) and were workers (57%). Moreover, 58.4% were imprisoned for drug offenses. Besides, 59.1% had opiate addiction, and only 45.6% had 30 minutes of physical activity per week (Table 1).

**Fig. 1:**
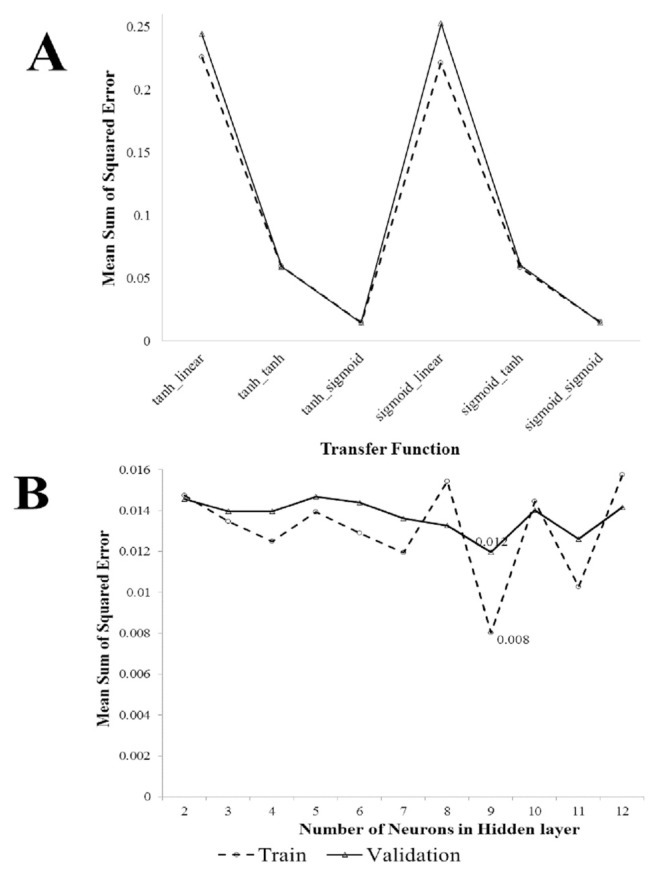
A) Reported mean square error for different transfer function s’ ANN models in both training and testing sets; B) Reported mean square error of selected ANN transfer function models with different hidden neurons in both training and testing sets.

In Fig. 1A, a combination of different functions with two neurons in the middle layer is used to determine the best function for connecting input, hidden, and output layers in the neural network. As it can be seen, the neural network with a hyperbolic tangent function used to connect the input and middle layers and a sigmoid function to connect the middle and output layers had the least mean sum of squared error in both the train and validation sets. Increasing the number of middle layer neurons to 9 could also reduce the sum of squared errors in both the train and validation sets to 8e-03 and 12e-03, respectively (Fig. 1B).

**Fig. 2:**
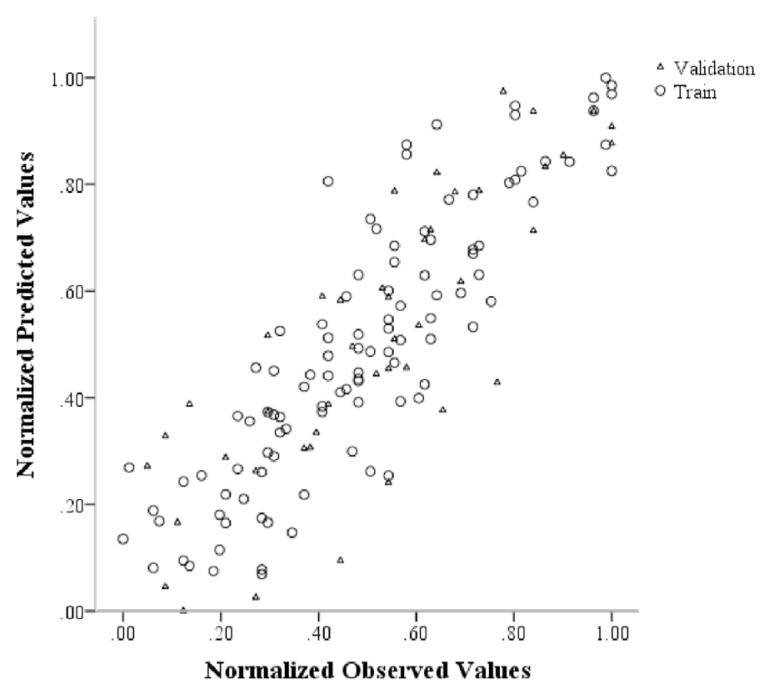
The normalized observed versus predicted GHQ scores or selected ANN transfer function in training and testing sets separately.

Finally, the optimal neural network performance (neural network with 9 neurons in the middle layer and hyperbolic tangent and sigmoid functions for connecting the input, middle, and output layers, respectively) is displayed in Fig. 2. The figure depicts the expected and actual standardized values for the train and validation sets. As it can be seen, expected and actual values had a direct linear relationship; therefore the neural network model has performed well in predicting prisoners’ general health by using variables such as age, education level, occupation, life and acceptance expectancy, cause of incarceration, type of drug misuse, and physical activity.

**Fig. 3:**
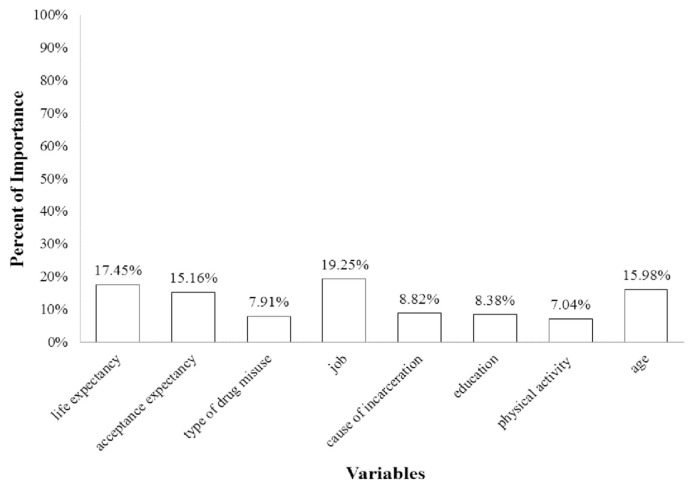
The variable importance from the selected Artificial Neural Network.

Figure 3 shows that from among the studied variables, occupation, life expectancy, age, and hope of acceptance were identified as the first most significant factors with 19.25, 17.45, 15.98, and 15.16 percentages, respectively, whereas the cause of incarceration, education level, type of drug misuse, and physical activity were the second most important factors with 8.82, 8.38, 7.91, and 7.04 percentages, respectively. As shown in Fig. 3, the importance of demographic variables in determining the general health of prisoners is close to 44%, followed by psychological variables with approximately 33%, and criminology variables with approximately 17%. The study also found that the physical activity of prisoners played a minor role in determining their general health.

## 4. Discussion

The findings of this study highlight the role of age, education level, occupation, life and acceptance expectancy, the cause of incarceration and the type of drug misuse, and physical activity in prisoners’ general health. One of the most significant findings of this study is the effect of occupation on prisoners’ health. Occupation and education are significant determinants of socioeconomic status. According to available evidence, an individual’s socioeconomic capital correlates with his/her perception of social support [32]. People with higher socioeconomic capital are less likely to be involved with psychosocial stresses as they enjoy internal capacities (higher intelligence and greater adaptability) or have access to external resources (greater welfare, better health facilities, or social power and influence) [2, 33–35]. Thus, they have a higher life expectancy than their peers and are likely to receive emotional social support or the hope of acceptance by their family and community [32].

Social support networks comprise individuals who provide support resources such as financial, instrumental, emotional, and informational support when needed [17]. The findings of various studies have shown the protective effect and moderating role of social support in the face of adverse events and coping with stress [36]. Social support creates conditions that make one feel loved, cared for, and valued when needed [17]. Therefore, it can be said that people who have higher emotional and social support have a higher expectancy and hope of acceptance by others [37]. The experience of emotional support can help build a positive attitude and develop a greater ability to cope with problems. As such, prisoners with the hope of acceptance in the family and community have a better prospect of the future and may choose effective strategies to manage challenges and stresses. Hence, they are less likely than other people to be inflicted by physical, mental, and psychological disorders [38]. On the other hand, deprivation of emotional and social support can reduce one’s ability to cope with stress and increases one’s willingness to engage in risky behaviors such as substance abuse or a tendency to use more dangerous substances [36]. Alongside this, the results of some studies have indicated that some motivations for drug use in prison entail pain reduction, avoidance of problems, and feelings of belonging and acceptance in the social network of other drug addicts in the prison [39, 40].

A significant and expected finding of the present study, also reported in other studies, concerns with the determinant role of age on prisoners’ health [41, 42]. Physical problems and disabilities are higher among older inmates than their non-prison counterparts [14]. Chronic diseases such as diabetes, heart attacks, hypertension, cognitive impairment, and liver disease are high in this group of prisoners [2, 14]. Besides, there is a higher incidence of psychological distress and mental problems in these individuals than in younger prisoners. However, factors that increase the risk of physical and mental illness in this age group may be related to the conditions of the individual before incarceration or because of the characteristics of the prison environment [2, 43, 44].

The relevance of the cause of incarceration or the type of crime to health corresponds with the results of previous studies. According to these results, more serious crimes and dangerous crimes related to acts of violence have a greater impact on physical and mental health than non-aggressive acts. It is because these criminals have to spend longer or sometimes all their lives in the judiciary and correctional systems and may even be condemned to retribution and death [9, 10]. On the other hand, stricter laws, excessive restrictions, and the increased time needed to determine the type of punishment (judge’s verdict), especially for the marginalized and underserved individuals, can be potential stressors, even for a mild charge. Also, inmates who have no previous incarceration history may endure greater stress and mental pressure due to being uncertain of or unfamiliar with the process of this particular system [16, 45]. According to some studies, the early years of incarceration are a dangerous stage for developing mental health disorders and depression [45, 46]. The absence of emotional and psychological support from family and society and the lack of opportunity or ability to cope with challenges and problems can also contribute to the development of these disorders.

### 4.1. Limitations

In this study, it was not possible to have access to a very large sample size because of the small target population. However, with the same volume of data, the performance of the neural network was appropriate, and it was able to identify the pattern between the data well. On the other hand, as in most similar studies, it was not possible to perform cohort studies and establish causal relationships because of the difficult conditions, the inability to follow individuals, and the sensitivity of participants. Moreover, the self-reporting nature of prisoners’ health information in this study can affect, as in most similar studies, the accuracy of the results. Therefore, if possible, it is recommended to consider prospective studies and to investigate the impact of other variables such as “duration have served a prison service” and “remaining duration in prison service” that may affected general health.

Experience of psychosocial pressures related to incarceration tends to be higher among the excluded and disadvantaged individuals, persons in very low or high age ranges, and individuals with specific crimes, and this increases the chances of health disorders in them. Consideration of the needs of prisoners, tailored to their individual, social, and emotional characteristics, especially in the early stages of their incarceration, can be useful in shaping the process of effective adjustment and choosing appropriate and effective strategies.

## Figures and Tables

**Table 1:** Demographic risk factors between responders.

Variable	N^a^ (%)
Age	36.58 ± 8.73
Optimism and hope for the future	
Very low	37 (24.8)
Low	41 (27.5)
High	32 (21.5)
Very high	39 (26.6)
Hope of acceptance	
Very low	43 (28.9)
Low	40 (26.8)
High	33 (22.1)
Very high	33 (22.1)
Education	
Illiterate	27 (18.1)
Primary/Secondary School	73 (49)
High School and Above	49 (32.9)
Job	
Unemployed	34 (22.8)
Worker	58 (57)
Non-worker	30 (20.1)
Cause of Incarceration	
Property	37 (24.8)
Persons	25 (16.8)
Drugs	87 (58.4)
Type Of Drug Misuse	
Non-Smoking	38 (25.5)
Smoking	23 (15.4)
Opioid	88 (59.1)
30 Minutes of Physical Activity Per Week	
Yes	68 (45.6)
No	81 (54.4)

a Data presented as mean ± SD or number (%).
